# Detection of Shiga toxin-producing *Escherichia coli* in ground beef using the GeneDisc real-time PCR system

**DOI:** 10.3389/fcimb.2012.00152

**Published:** 2012-12-20

**Authors:** Pina M. Fratamico, Lori K. Bagi

**Affiliations:** Eastern Regional Research Center, Agricultural Research Service, US Department of AgricultureWyndmoor, PA, USA

**Keywords:** GeneDisc, real-time PCR, STEC, virulence genes, O-group, detection, isolation, *E. coli* O157:H7

## Abstract

*Escherichia coli* O157:H7 and certain non-O157 Shiga toxin-producing *Escherichia coli* (STEC) serogroups have emerged as important public health threats. The development of methods for rapid and reliable detection of this heterogeneous group of pathogens has been challenging. GeneDisc real-time PCR assays were evaluated for detection of the *stx*_1_, *stx*_2_, *eae*, and *ehxA* genes and a gene that identifies the O157 serogroup followed by a second GeneDisc assay targeting serogroup-specific genes of STEC O26, O45, O91, O103, O111, O113, O121, O145, and O157. The ability to detect the STEC serogroups in ground beef samples artificially inoculated at a level of ca. 2–20 CFU/25 g and subjected to enrichment in mTSB or buffered peptone water (BPW) was similar. Following enrichment, all inoculated ground beef samples showed amplification of the correct set of target genes carried by each strain. Samples inoculated with STEC serogroups O26, O45, O103, O111, O121, O145, and O157 were subjected to immunomagnetic separation (IMS), and isolation was achieved by plating onto Rainbow agar O157. Colonies were confirmed by PCR assays targeting *stx*_1_, *stx*_2_, *eae*, and serogroup-specific genes. Thus, this work demonstrated that GeneDisc assays are rapid, sensitive, and reliable and can be used for screening ground beef and potentially other foods for STEC serogroups that are important food-borne pathogens worldwide.

## Introduction

In addition to *Escherichia coli* O157:H7, numerous non-O157 Shiga toxin-producing *E. coli* (STEC) serogroups have caused outbreaks and severe illness, including hemorrhagic colitis (HC) and hemolytic uremic syndrome (HUS) similar to STEC O157. Although *E. coli* O157:H7 causes many of the STEC-related outbreaks in the US, it is estimated that non-O157 STEC cause more than twice the number of infections overall compared to *E. coli* O157:H7 (Scallan et al., 2011). Furthermore, non-O157 STEC cause the majority of STEC-associated infections in some countries (Johnson et al., 2006). Data from the Centers for Disease Control and Prevention indicate that STEC serogroups O26, O45, O103, O111, O121, and O145 cause the majority of cases of illness due to non-O157 STEC in the US and are important STEC serogroups in other countries, as well (Brooks et al., 2005; Johnson et al., 2006; Gyles, 2007). These STEC serogroups are referred to as the “top six” or “big six” serogroups, and major outbreaks of HC and HUS associated with these non-O157 STEC have been reported (Bettelheim, 2007; Mathusa et al., 2010; Schaffzin et al., 2012). Other STEC serogroups including O91 and O113 have also been linked to cases and outbreaks of HC and HUS and are important STEC in many countries (Paton et al., 1999; Johnson et al., 2006; Bettelheim, 2007; EFSA, 2009; Mellmann et al., 2009).

It is likely that highly pathogenic STEC have acquired genetic elements encoding specific virulence factors through lateral gene transfer to a greater extent than strains with lesser virulence. STEC carry phage-encoded Shiga toxin genes, *stx*_1_ and/or *stx*_2_ or various genetic subtypes of these genes. STEC also possess a pathogenicity island, known as the locus of enterocyte effacement (LEE), which encodes proteins necessary for the formation of attaching and effacing lesions, including intimin (Eae, an outer membrane protein), a translocated intimin receptor (Tir), a type III secretion apparatus, and effector proteins translocated by the secretion system (Gyles, 2007; Bolton, 2011). The precise role of a plasmid-encoded enterohemolysin, EhxA, in STEC virulence remains to be resolved; however, the presence of the gene is a defining diagnostic epidemiological marker in outbreak strains and strains that cause severe disease (Schmidt and Karch, 1996; Boerlin et al., 1999; Brooks et al., 2005). Analysis of STEC belonging to different serotypes showed that there was a significant association between the presence of the *eae* gene and bloody diarrhea and the *stx*_2_ gene with HUS (Brooks et al., 2005). STEC O113 and O91 that have caused outbreaks and severe disease lack the *eae* gene (Paton et al., 1999; Mellmann et al., 2009); however, these serogroups carry other genes, including *saa* (STEC autoagglutinating adhesion) (Paton et al., 2001; Gyles, 2007) that may allow attachment to host cells. A large outbreak that occurred in 2011 in Germany linked to sprouts was caused by *E. coli* O104:H4 that carried the *stx*_2_ gene; however, the strain was an enteroaggregative *E. coli* (EAEC) that also carried adherence genes located on pAA, the large virulence plasmid of EAEC, that allowed attachment to cells (Bielaszewska et al., 2011), and the strain did not carry the *eae* gene. Thus, strains that lack *eae* may cause severe disease, and public health authorities should remain vigilant for these emerging STEC serogroups.

Similar to STEC O157, cattle and other ruminants are reservoirs for non-O157 serotypes, and surveys have demonstrated their presence in samples from cattle carcasses, retail beef, and raw milk, as well as other food (Hussein, 2007; Mathusa et al., 2010; Bosilevac and Koohmaraie, 2011). Food of bovine origin, food or water contaminated with bovine feces, and animal contact are important vehicles and modes of transmission of STEC infections (Kaspar et al., 2010). Due to the serious health concerns of non-O157 STEC infections, particularly due to specific *E. coli* serogroups, the United States Department of Agriculture's Food Safety and Inspection Service (USDA FSIS) declared the top six non-O157 STEC as adulterants in raw, non-intact beef products (Anonymous, 2011), and regulatory testing for these STEC serogroups began in June, 2012. Thus, there is a need for rapid methods that can be used for regulatory testing and for detection of the top six STEC serogroups by the food industry. There have been numerous reports on PCR-based methods for detection of food-borne pathogens, including STEC. Many of these PCR-based methods require the preparation of the individual components used for the reaction mixture, lengthy and cumbersome DNA extraction procedures, do not employ multiplexed detection, or do not include an internal amplification control (Hoorfar and Cook, 2003). A method that has flexibility is sensitive and is less time consuming and cumbersome would be more amenable for use by regulatory agencies and the food industry. One such method with these advantages is the GeneDisc Rapid Microbiology System manufactured by Pall GeneDisc Technologies that uses real-time PCR technology. The flexibility of the GeneDisc platform allows targeting specific STEC serogroups of interest and STEC virulence genes. The objective of the current study was to compare two different enrichment media and evaluate GeneDisc real-time PCR assays to detect nine important STEC serogroups: STEC O26, O45, O91, O103, O111, O113, O121, O145, and O157 in ground beef.

## Materials and methods

### Bacterial strains and growth conditions

STEC strains used in this study are listed in Table 1 and were stored at −80°C in tryptic soy broth (TSB) (Becton Dickinson and Co., Sparks, MD) containing 10% glycerol. The bacteria were grown overnight in TSB at 37°C, and dilutions of the cultures were made in 0.1% sterile peptone (Bacto™ Peptone, Becton Dickinson and Co.).

**Table 1:** **Serotypes, source, and virulence gene profiles of STEC strains used in this study**.

**Serotype and strain**	**Source^a^**		***stx*_1_**	***stx*_2_**	***eae***	***ehxA***
O26:H11 05-6544	PHAC	human	+^b^	−^b^	+	+
O45:H2 05-6545	PHAC	human	+	−	+	+
O91:H21 85-489	PHAC	human	−	+	−	+
O103:H25 03-2444	PHAC	human	+	−	+	+
O111:NM 00-4748	PHAC	human	+	+	+	−
O111:NM 98-8338	PHAC	human	+	−	+	+
O111:H8 01387	FDA	human	+	−	+	+
O111:H8 SJ14	CDC	human	+	+	+	+
O111:NM SJ15	CDC	human	+	+	+	+
O111:+^c^ E05	University of Minnesota	cattle	+	−	+	+
O113:H21 04-1450	PHAC	human	−	+	−	+
O121:NM 03-4064	PHAC	human	−	+	+	+
O145:NM 03-4699	PHAC	human	+	−	+	+
O157:H7 380-94	FSIS	human	+	+	+	+

a PHAC, Public Health Agency of Canada, Winnipeg, Manitoba, Canada; FDA, Food and Drug Administration, Center for Food Safety and Applied Nutrition, College Park, MD; CDC, Centers for Disease Control and Prevention, Atlanta, GA; Dr. Francisco Diez-Gonzalez, University of Minnesota, St. Paul, MN; FSIS, Food Safety and Inspection Service, Athens, GA.

b “+”, positive; “−”, negative.

c The “+” indicates that the fliC gene was amplified by PCR in this strain; however, the restriction fragment length polymorphism pattern did not match with any of the known H types.

### Inoculation and enrichment of ground beef

Ground beef was purchased from local supermarkets. One package of ground beef was divided into 25-g portions that were placed in sterile filter Stomacher bags (Seward Ltd., West Sussex, UK). The meat was inoculated with 1 mL of a diluted culture to achieve a target inoculum level of ca. 2–20 CFU/25 g, and then either 225 mL of mTSB enrichment medium or buffered peptone water (BPW) (Oxoid Ltd., Basingstoke, Hampshire, England) was added, followed by pummeling in a Stomacher apparatus (Seward Ltd.) for 1 min. The CFU/mL of the inoculum was determined by plating the cell dilutions onto tryptic soy agar (TSA) and enumerating the colonies after overnight growth at 37°C. An uninoculated control ground beef sample was included with each set of samples. mTSB was prepared as described previously (Fratamico et al., 2011) by adding cefsulodin (10 mg/L) and vancomycin (16 mg/L) (Sigma-Aldrich Corp., St. Louis, MO), and samples were incubated static for 6 h at 37°C. After this 6-h enrichment step, bile salts (1.5 g/L), rifampicin (2.0 mg/L), and potassium tellurite (1.0 mg/L) were added (Sigma-Aldrich Corp., St. Louis, MO) to the samples, and incubation was continued static for 18 h at 42°C. Enrichment in BPW (pre-warmed to 42°C) was performed at 42°C for 18 h as recommended by the GeneDisc manufacturer.

### DNA extraction

DNA extraction was performed using the GenDisc lysis reagent (Pall GeneDisc Technologies, Bruz, France). Following enrichment in mTSB or BPW, 50 μL of the enrichment were transferred to a lysis tube provided with the lysis solution and heated at 100°C for 10 min. The extracted DNA was either transferred to a GeneDisc for real-time PCR or stored at −20°C for testing at a later date.

### Genedisc real-time PCR assay

Mastermix (37 μL) provided with the GeneDisc kits and 37 μL of template DNA were mixed in a sterile tube, and then 72 μL of the mixture for each sample were placed into the central reservoir of one of the GeneDisc sectors. Vacuum was applied using a pump provided with the GeneDisc Cycler to pull the mixture into the sector microchambers around the perimeter of the GeneDisc, which contained dessicated primers and probes labeled with either 6-FAM or ROX. The GeneDisc was inserted into the GeneDisc Cycler, following the manufacturer's instructions, and the reaction was started. The PCR consisted of 45 cycles, and the assay was completed in ca. 75 min. Two GeneDiscs were employed. The first disc (STEC and *E. coli* O157 GeneDisc) was composed of six sectors, consisting of real-time multiplex PCR assays targeting *stx*_1_, *stx*_2_, *eae*, *ehxA*, and O157. The microwells in each sector consisted of the following multiplex PCR assays with primers and labeled probes: microwell 1—negative control (FAM) and inhibition control (ROX), microwell 2—*ehxA* (FAM) only, microwell 3—*stx*_2_ (FAM) and *stx*_1_ (ROX), microwell 4—*stx*_2_ (FAM) and *stx*_1_ (ROX), microwell 5—O157 (FAM) and *eae* (ROX), and microwell 6—O157 (FAM) and *eae* (ROX). The second (GeneDisc 9 EHEC Identification) was also composed of six sectors and multiplex PCR assays targeted O-group-specific genes of serogroups O26, O45, O91, O103, O111, O113, O121, O157, and O145. The microwells in each sector consisted of: 1—only O145 (FAM), 2—negative control (FAM) and PCR inhibition control (ROX), 3—O157 (FAM) and O111 (ROX), 4—O26 (FAM) and O103 (ROX), 5—O91 (FAM) and O113 (ROX), and 6—O121 (FAM) and O45 (ROX). The decision support software installed in the GeneDisc Cycler processes the results, which can be exported as a table and are also presented as graphic images of the amplification curves.

### Immunomagnetic separation and plating and isolation on rainbow agar O157

The O26, O45, O103, O111, O121, O145, and O157 enrichments were also subjected to immunomagnetic separation (IMS) using Dynabeads EPEC/VTEC O26, O103, O111, O145, and O157 (Fratamico et al., 2011). For STEC O45 and O121, streptavidin-coated beads (Invitrogen Corp., Carlsbad, CA) were prepared using biotinylated polyclonal antibodies against *E. coli* serogroups O45 and O121 as described previously (Fratamico et al., 2011). IMS for STEC O91 and O113 was not performed. The beads–bacteria complexes were plated onto Rainbow Agar O157 (Biolog, Inc., Hayward, CA) containing 0.8 mg/L of potassium tellurite and 10 mg/L of novobiocin, and dilutions of enrichments from beef samples that had been inoculated with STEC O91 and O113 were plated directly onto the same agar. Two to five presumptive colonies were picked from the plates and confirmed by conventional PCR assays targeting *stx*_1_, *stx*_2_, *eae*, and the specific O-group *wzx* or *wbdI* genes (Fratamico et al., 2011). The primers and probe targeting the *E. coli* O157 *wzy* gene were described previously (Fratamico and DebRoy, 2010).

## Results and discussion

In the US, *E. coli* O157:H7 and the top six non-O157 STEC (O26, O45, O103, O111, O121, and O145) cause the majority of the food-borne illnesses and outbreaks; however other emerging STEC serogroups including O113 and O91 have caused outbreaks and serious illness in the US and in other countries (Paton et al., 1999; Brooks et al., 2005; Johnson et al., 2006; Bettelheim, 2007; Mellmann et al., 2009). Cattle are an important reservoir for STEC O157:H7, as well as other STEC serogroups, and in September 2011, the FSIS announced that the top six non-O157 STEC would be declared as adulterants in beef similar to O157:H7. Thus, the availability of rapid screening methods for STEC of public health concern is critical. We evaluated the GeneDisc real-time PCR system developed by Pall GeneDisc Technologies. The GeneDisc for detection of specific O serogroups (O26, O45, O91, O103, O111, O113, O121, O145, and O157) was developed for evaluation in our laboratory in consultation with the manufacturer. The technology allows simultaneous detection of multiple targets by real-time PCR. The first GeneDisc screening assay targeted *stx*_1_, *stx*_2_, *ehxA*, *eae*, and O157, and the second was used to determine the presence of nine STEC O groups targeting serogroup-specific regions within the O-antigen gene clusters of these STEC. The first assay could be used to detect the presence of *E. coli* O157. A GeneDisc assay that contained primers and probe targeting the *fli*C_H7_ gene has also been described (Beutin et al., 2009). These investigators evaluated GeneDisc assays for detection of STEC O26, O103, O111, O145, and O157 using DNA from colonies and from pure and mixed cultures. One GeneDisc assay targeted *rfb*E_O157_, *stx*_1_/*stx*_2_, *fli*C_H7_, and *eae*, and a second GeneDisc targeted *wzx*_O26_, *wbd*I_O111_, *ihp*I_O145_, *wzx*_O103_, and *ihp*I_O157_. The assays were specific for the target organisms, and the sensitivity was <10 bacteria/sector. However, the *stx*_1_/*stx*_2_ assay required >100 bacteria for a positive result. Strains carrying the *stx*_2f_ variant and the *eae* rho variant were not detected. The sequences of the primers and probes in the GeneDisc assays used in the current study are proprietary; however, based on discussions with the manufacturer, the *stx*_2f_ and *eae* rho variants are not detected with the assays. The *stx* and *eae* subytpes of the strains tested in this study were not determined; however, PCR assays using primers described by Fratamico and DebRoy (2010) and Fratamico et al. (2011) were used to determine if the strains carried *stx*_1_, *stx*_2_, and *eae*.

In the current study, we compared enrichment in BPW (as recommended by GeneDisc) and mTSB for STEC detection in ground beef. mTSB contained selective agents, and the total enrichment time was 24 h (6 h at 37°C and 18 h at 42°C after addition of three other selective agents). BPW does not contain selective agents and enrichment was for 18 h at 42°C. PCR results with mTSB and BPW were similar. Uninoculated ground beef samples were negative for all of the target genes. The cycle threshold (Ct) values for samples enriched in mTSB ranged from 18.5 ± 1.0 to 26.9 ± 4.8 (Table 2). The Ct values for PCR assays using DNA extracted from samples enriched in BPW ranged from 18.6 to 26.3 ± 5.9 (Table 3). Target genes in all of the STEC strains tested were correctly identified, and there were no notable differences in Ct values among the different target genes amplified. Some variability in initial inoculum levels, strain to strain variability in growth, and DNA extraction may have accounted for some of the differences observed in Cts. The standard deviations were based on results of at least three different experiments, except for STEC O111 in which there were three experiments done with strain 00-4748, which was negative for *ehxA*, and only one experiment each for strains 98-8338, 01387, SJ14, SJ15, and E05. Each experiment consisted on one sample inoculated with each serogroup. Several strains were used for STEC O111 because it was more difficult to isolate this serogroup on the Rainbow Agar O157 plates compared to the other strains, and we wanted to verify if there was strain to strain variation in the ability to identify STEC O111 colonies on Rainbow Agar O157. The STEC O91 and O113 strains tested lacked the *eae* gene, as expected. Several other O91 and O113 strains in our culture collection were also tested to determine the presence of the *eae* gene, and all were negative (data not shown).

**Table 2:** **Average cycle thresholds ± STDEV of GeneDisc assays using mTSB for enrichment of ground beef samples**.

**Serogroup-strain^a^**	***stx*_1_**	***stx*_2_**	***eae***	***ehxA***	**O157^b^**	**O group^b^**
O26	24.8 ± 0.8	–	26.5 ± 0.6	24.7 ± 0.8	–	25.3 ± 1.1
O45	22.1 ± 3.4	–	24.0 ± 3.1	22.4 ± 2.9	–	22.7 ± 2.6
O91	–	21.0 ± 1.5	–	20.4 ± 1.5	–	20.8 ± 1.3
O103	20.0 ± 2.9	–	21.8 ± 2.3	20.5 ± 2.0	–	21.8 ± 2.4
O111						
00-4748	25.0 ± 4.4	25.2 ± 4.2	26.9 ± 4.8		–	26.0 ± 4.7
98-8338	23.6	–	24.3	23.0	–	23.5
01387	25.2	–	25.7	24.0	–	25.4
SJ14	21.4	22.0	22.2	20.6	–	20.9
SJ15	21.0	19.7	23.4	20.0	–	24.4
E05	20.9	–	21.4	19.7	–	22.2
O113	–	18.8 ± 0.8	–	19.1 ± 0.9	–	18.5 ± 1.0
O121	–	23.2 ± 2.1	24.2 ± 2.3	22.5 ± 2.1	–	21.7 ± 1.9
O145	20.3 ± 3.4	–	22.6 ± 3.9	21.0 ± 3.2	–	21.9 ± 5.5
O157	18.9 ± 0.4	19.5 ± 0.2	20.9 ± 0.5	20.0 ± 0.7	19.9 ± 0.4	21.0 ± 0.6

a Results represent averages from at least three separate experiments except for O111 strains 98-8338, O1387, SJ14, SJ15, and E05 for which only one experiment was performed.

b Results in the column labeled O157 represent data from the STEC and E. coli O157 GeneDisc that targeted virulence genes, as well as O157. The column labeled O group shows data from the GeneDisc 9 EHEC Identification that targeted the 9 O serogroups.

**Table 3:** **Average cycle thresholds ± STDEV of GeneDisc assays using BPW for enrichment of ground beef samples**.

**Serogroup-strain^a^**	***stx*_1_**	***stx*_2_**	***eae***	***ehxA***	**O157^b^**	**O group^b^**
O26	22.7 ± 3.2	–	24.8 ± 4.1	23.2 ± 3.2	–	22.5 ± 3.1
O45	20.9 ± 3.7	–	23.6 ± 4.3	21.6 ± 3.7	–	22.6 ± 3.4
O91	–	22.7 ± 3.7	–	22.0 ± 4.5	–	22.4 ± 3.5
O103	22.2 ± 3.1	–	23.8 ± 3.2	22.4 ± 3.2	–	23.3 ± 2.7
O111						
00-4748	22.8 ± 4.6	23.0 ± 6.0	23.2 ± 2.6	–	–	21.9 ± 4.2
98-8338	21.7	–	22.7	21.7	–	20.4
01387	21.1	–	22.2	20.7	–	20.3
SJ14	20.0	20.8	22.0	20.6	–	20.3
SJ15	21.4	18.6	23.2	19.0	–	21.2
E05	20.0	–	21.2	19.4	–	19.4
O113	–	20.7 ± 2.8	–	21.1 ± 3.2	–	21.2 ± 3.0
O121	–	24.6 ± 6.7	26.3 ± 5.9	24.2 ± 5.9	–	24.6 ± 4.4
O145	22.9 ± 3.7	–	25.2 ± 4.7	23.5 ± 3.9	–	25.3 ± 3.7
O157	18.8 ± 2.3	19.2 ± 2.3	19.6 ± 0.9	20.0 ± 1.8	19.1 ± 1.0	20.9 ± 3.3

a Results are averages from at least three separate experiments except for O111 strains 98-8338, O1387, SJ14, SJ15, and E05 for which only one experiment was performed.

b Results in the column labeled O157 represent data from the STEC and E. coli O157 GeneDisc that targeted virulence genes, as well as O157. The column labeled O group shows data from the GeneDisc 9 EHEC Identification that targeted the 9 O serogroups.

Screening assays based on detection of *stx* and *eae* followed by PCR assays targeting genes in the O-antigen gene clusters of the top six non-O157 STEC serogroups are useful for detection of these serogroups; however, since some serogroups, including STEC O91 and O113 lack the *eae* gene, they would not be identified using screening assays that rely on samples to be positive for both *stx*_1_/*stx*_2_ and *eae*. Therefore, an assay that targets additional virulence genes such as *ehxA* and O-group-specific genes of the *eae*-negative STEC, such as serogroups O91 and O113, are useful for detection of these pathogens. A number of assays targeting STEC virulence genes and O-group-specific genes have been reported. Perelle et al. (2004) developed real-time PCR assays to detect STEC serogroups (O26, O55, O91, O103, O111, O113, O145, and serotype O157:H7). The PCR assays that were designed targeted the *stx*_1_, *stx*_2_, and *fli*C_H7_ genes, as well as O-group-specific genes. These same investigators used a PCR-ELISA (enzyme-linked immunosorbent assay) targeting *stx*_1_ and *stx*_2_ for initial screening of food samples, and samples were further tested by a multiplex PCR assay targeting O-group-specific genes, the O103 *eae* gene, and an O-island gene of O145 (Perelle et al., 2007). This was followed by PCR assays to identify the specific O-group and *stx* type, and this method was used to screen raw milk and minced meat samples for STEC O26, O103, O111, O145, and O157. The results revealed that ca. 2.6 and 4.8% of minced meat and raw milk samples, respectively, were contaminated with the targeted STEC. This protocol is rather arduous and time consuming, and use of a method such as the GeneDisc system targeting all of the genes of interest in an easy to use format would be more rapid and less laborious.

It is important to recognize that detection of specific genes by PCR in food samples does not necessarily imply that the genes are carried by the same bacterium and that the bacterium is viable; therefore, isolation of the target pathogen is necessary. There are no commercially available plating media that are very suitable for differentiation and isolation of *E. coli* O157:H7 and important non-O157 STEC serogroups. Furthermore, there are selective agents used in agar media, which may inhibit some STEC strains. We described the use of Rainbow Agar O157 for isolation of non-O157 STEC in an earlier report (Fratamico et al., 2011); therefore, this agar was also used in current study. The typical color of the non-O157 STEC colonies on Rainbow Agar O157 was purple, magenta/mauve, blue-violet, gray, pink with dark pink center, and violet/light purple, for *E. coli* O26, O45, O103, O111, O121, and O145, respectively. *E. coli* O157:H7 appears as black or dark gray colonies on this agar. For the STEC that were subjected to IMS, typically 2–5 colonies were picked for confirmation by the PCR. *E. coli* O91 and O113 enrichments were plated directly without performing IMS; typical O91 and O113 colonies were blue-gray and cream in color, respectively. *E. coli* O91 and O113 colonies from enrichments that were not subjected to IMS were more difficult to identify on Rainbow Agar O157 due to the higher level of background colonies that were present; therefore, no colonies were picked for confirmation. Thirty-one to 100 percent of the presumptive STEC O26, O45, O103, O111, O121, O145, and O157 colonies picked from Rainbow Agar O157 were confirmed as the correct STEC serogroup, and generally the isolation rate was similar with colonies picked from mTSB and BPW enrichments (Table 4). The correct STEC O121 and O145 colonies were somewhat easier to pick and confirm from mTSB (100 vs. 55% confirmed for O121, respectively, and 90 vs. 31% for O145) compared to BPW; however, more work must be done to validate this observation.

**Table 4:** **Number (percentage) of colonies confirmed by real-time PCR assays out of total presumptive colonies picked from Rainbow Agar O157**.

**STEC serogroup**	**mTSB**	**BPW**
O26	8/10 (80%)	11/11 (100%)
O45	7/9 (78%)	12/12 (100%)
O91^a^	Not available	Not available
O103	9/9 (100%)	11/11 (100%)
O111	9/24 (37.5%)	20/39 (51%)
O113^a^	Not available	Not available
O121	9/9 (100%)	6/11 (55%)
O145	9/10 (90%)	4/13 (31%)
O157	7/9 (78%)	7/7 (100%)

a IMS was not performed on samples inoculated with STEC O91 and O113; therefore, colonies were not picked for confirmation by PCR.

Results of the current study demonstrate that the GeneDisc real-time PCR technology is rapid and simple to use, and it is a promising method for testing for STEC in food samples. The versatility of the GeneDisc system is evidenced by reports on the use of a GeneDisc array for detection of various STEC virulence and O-group- and H-group-specific genes (Bugarel et al., 2010a,b), for genotyping *Salmonella* (Bugarel et al., 2011), and for detection and genotyping of *Clostridium botulinum* (Fach et al., 2011; Sevenier et al., 2012; Woudstra et al., 2012). New types of assays can be designed by incorporating primers and probes for new gene targets. In the future, GeneDisc assays can be designed for detection of other STEC virulence genes and emerging STEC serogroups, including O104, O55, or others. The current study demonstrated that GeneDisc assays for STEC detection are rapid, simple, and accurate and can be used for screening for these pathogens in ground beef and potentially other foods, as well.

### Conflict of interest statement

The authors declare that the research was conducted in the absence of any commercial or financial relationships that could be construed as a potential conflict of interest.
